# The impacts of mind-wandering on flow: Examining the critical role of physical activity and mindfulness

**DOI:** 10.3389/fpsyg.2022.674501

**Published:** 2022-07-25

**Authors:** Yu-Qin Deng, Binn Zhang, Xinyan Zheng, Ying Liu, Xiaochun Wang, Chenglin Zhou

**Affiliations:** ^1^Institute of Sports Science, Nantong University, Nantong, China; ^2^School of Psychology, Shanghai University of Sport, Shanghai, China; ^3^School of Kinesiology, Shanghai University of Sport, Shanghai, China

**Keywords:** mind-wandering, physical activity, mindfulness, flow, mediation analysis

## Abstract

**Background:**

Individuals with mind-wandering experience their attention decoupling from their main task at hand while others with flow experience fully engage in their task with the optimum experience. There seems to be a negative relationship between mind-wandering and flow. However, it remains unclear to what extent mind-wandering exerts an impact on flow. And it is also elusive whether physical activity and mindfulness, which are as important factors that affected individuals’ attentional control and psychological health, are beneficial in explaining the association between mind-wandering and flow. The current study investigated the relationship between mind-wandering and flow, and the potential mediation effects of physical activity and mindfulness in this association.

**Methods:**

A cross-sectional exploratory study design, including multiple scales such as the Mind-Wandering Questionnaire (MWQ), the International Physical Activity Questionnaire Short Form (IPAQ), Mindfulness Attention and Awareness Scale (MAAS), and the Short Dispositional Flow Scale (S-DFS) was applied. Descriptive statistics and bivariate correlation coefficients were applied in the analysis of these data. A multiple mediation model was used to examine the relationships between mind-wandering, flow, physical activity, and mindfulness.

**Results:**

Mind-wandering was inversely associated with physical activity, mindfulness and flow, respectively; and flow was positively related to physical activity and mindfulness, respectively. Moreover, multiple mediation results demonstrated that physical activity and mindfulness, respectively, mediated the relationship between mind-wandering and flow.

**Conclusion:**

These findings are helpful to understand how our minds attend to the present moment, and the crucial roles of physical activity and mindfulness in the association between mind-wandering and flow. An implication of these is the possibility that the effective strategies aimed at enhancing both the levels of physical activity and mindfulness are needed to reduce the negative impact of mind-wandering on flow.

## Introduction

Mind-wandering is a ubiquitous phenomenon encompassing specific aspects of consciousness in which the mind loses focus on the present moment and begins to generate spontaneous thoughts (Smallwood and Schooler, 2015). Abundant experimental evidence has demonstrated that mind-wandering has detrimental effects on tasks that require undivided attention (i.e., reading comprehension and tests of cognitive performance), and self-reported depressive mind (Mooneyham and Schooler, 2013; Deng et al., 2014). On the other hand, mind-wandering contributes to creativity and innovation, as it enables the establishment of new connections between previous and potential experiences (Mooneyham and Schooler, 2013). Self-generated thoughts can be conceptualized as involving a conscious state of internal attention decoupled from current perception (i.e., not based on perceptual input) (Schooler et al., 2011). They are dynamic, and individual can intermittently monitor their content explicitly through meta-awareness (Smallwood and Schooler, 2015). According to the Context Regulation Hypothesis, mind-wandering can be regulated to generate optimal cognition to meet external demands (i.e., task performance) (Smallwood and Andrews-Hanna, 2013; Smallwood and Schooler, 2015; Thomson et al., 2015; Smallwood et al., 2021). Thus, it is considered to be a form of adaptive attention control facilitating adjustment to the environment or creative problem solving (Kam et al., 2013; Smallwood and Schooler, 2015). Given the costs and benefits of mind-wandering, the identification of its effects mind-wandering and exploration of its relationships with other variables are important. Our previous study demonstrated that self-control and mindfulness sequentially mediate the relationship between mind-wandering and metacognition (Deng et al., 2019). The finding makes an important contribution to our understanding of the characteristics of information processing in our mind. However, more research is required to determine the effects of mind-wandering, and to provide a theoretical basis for the understanding of psychological adjustment.

Unlike mind-wandering, flow is viewed as a special type of attention in which individuals are fully attuned to the present moment to attain the greatest effectiveness (Nakamura and Csikszentmihalyi, 2014). A recent study showed that positively perceived feedback boosted billiard players’ flow experience, enabling them to more mindfully and flexibly self-regulate their focus on the present moment, with reduced attention to distractions that could impede their performance. Thus, flow, as a subjective experience of “deep and effortless concentration,” entails reduced mind-wandering and increased mindfulness (Marty-Dugas and Smilek, 2018; Lambert and Csikszentmihalyi, 2019). However, the mechanisms underlying the negative relationship between flow and mind-wandering remain unclear. According to the attentional model, the experience of flow involves the narrowing of attention and enhancement of moment-to-moment orientation, whereas mind-wandering involves the broadening of attention and reduction of moment-to-moment orientation (Dane, 2011; Dust, 2015); both states thus rely on attention control. Mind-wandering has been referred to as the “dynamic redistribution of attention resources,” in which individuals’ attention drifts away from external tasks to ongoing internal thoughts (Smallwood and Andrews-Hanna, 2013; Smallwood and Schooler, 2015; Thomson et al., 2015; Smallwood et al., 2021). Individuals with better attention control tend to have enhanced meta-awareness and regulation of self-generated thoughts (Schooler et al., 2011). Flow leads to the efficient allocation of the limited attentional resources, resulting in effortless concentration, and improvement of task performance (Nakamura and Csikszentmihalyi, 2014; Harris et al., 2017). Few studies, however, have explored how spontaneous thoughts influence the flow experience.

Mind-wandering and flow both enhance creativity. Especially during the performance of undemanding tasks, mind-wandering allows for the generation of more spontaneous thoughts and ebb and flow of attention (Schooler et al., 2014; Williams et al., 2018; Zedelius and Schooler, 2020); similarly, greater flow experiences have been found to be related significantly to greater degrees of creativity (MacDonald et al., 2006; Schutte and Malouff, 2020). Thus, mind-wandering might be expected to predict flow, but more research is needed to examine this potential relationship and its mediators. Mind-wandering occurs with high frequency, occupying about half of the individuals’ waking hours, and is thus, a prevalent and important experience in daily life (Smallwood and Schooler, 2015). On the other hand, flow is rarely attained, but plays a beneficial role in routine task performance (Nakamura and Csikszentmihalyi, 2014). Individuals experiencing flow tend to effectively cope with and enjoy the task at hand, and ultimately to have improved their quality of life. Knowledge of the relationship between mind-wandering and flow, and the mechanisms underlying it would provide insight on how to make spontaneous thoughts more positive and adaptive, have more optimal experiences in our daily lives, and understand the characteristics of our mental activity more deeply.

Based on the previous research, mindfulness may be involved in the association between mind-wandering and flow. Mindfulness, as the present-being experience that induces self-regulation of attention and awareness (Karunamuni and Weerasekera, 2019) has beneficial effects on attentional control and psychological health (Karunamuni and Weerasekera, 2019; Prakash et al., 2020). The Mindful Awareness Attention Scale (MAAS) has been developed for the assessment of dispositional mindfulness. MAAS scores correlate negatively with multiple self-reported mind-wandering, clarifying that these constructs are opposite (Mrazek et al., 2012; Stawarczyk et al., 2012). Individuals with lower MAAS scores are more likely to report higher frequencies of mind-wandering during driving or the performance of demanding cognitive tasks (Burdett et al., 2016; Ju and Lien, 2018). Conversely, greater mindfulness correlates with less mind-wandering and a greater frequency of flow (Deng et al., 2014; Marty-Dugas and Smilek, 2018; Lambert and Csikszentmihalyi, 2019; Xie, 2021). Mindfulness and flow are both characterized by the strong focus of attention at the present moment (Wright et al., 2006; Šimleša et al., 2018). Through the facilitation of attentional control, mindfulness can help individuals be more aware of current thoughts and goal-directed actions, and guide them from mind-wandering to a focus on the current task (Smallwood and Schooler, 2015; Prakash et al., 2020). Consequently, mindfulness may mediate the relationship between mind-wandering and flow, but further research is needed to test this hypothesis.

Physical activity has beneficial effects on attentional control and psychological health (Plante and Rodin, 1990; Winneke et al., 2012; Fortier and Morgan, 2021), and has been shown to be associated closely with mind-wandering and flow. Current spontaneous thoughts have been demonstrated to be significantly associated with moderate-to-vigorous physical activity (Fanning et al., 2016) while physical activity has been shown to influence the ability to exert flexible cognitive control, which is closely related to the mind-wandering (Buckley et al., 2014). Higher levels of physical activity are also more likely to contribute to the experience of flow, especially in athletes and inactive individuals (Jackson and Eklund, 2002; Elbe et al., 2010). Among athletes, greater task orientation (reflecting clearer goal and sensitive to control over the activity) has been shown to be related closely to flow (Stavrou et al., 2015). In addition, individuals engaging in recreational physical activity have been found to experience more flow-like episodes correlated with greater situational involvement; these experiences and feelings are enjoyable (Decloe et al., 2009). Inactive individuals have been found to experience flow after long-term physical activity interventions (Elbe et al., 2010). All of these findings imply that physical activity could be as important factors in the flow experience. Thus, physical activity may mediate the association between mind-wandering and flow.

The current study was conducted to investigate correlations among mind-wandering, mindfulness, physical activity, and flow, and to examine the mediating effects of mindfulness and physical activity on the relationships between mind-wandering and flow. The hypothesis was that mind-wandering would be related to lower levels of mindfulness and physical activity, which in turn would be related to lower levels of flow. A multiple mediator analysis would then be performed to compare the different mediating effects of mindfulness and physical activity on the associations between mind-wandering and flow.

## Materials and methods

### Participants

The present research was approved by relevant institutional ethics committees. Anonymous, self-report questionnaires (described later) were distributed to a sample of 465 healthy college students in China. The participants completed the questionnaires with a pen. A total of 36 participants did not completely fill in these measurements. Finally, data collected from 429 Chinese college students (103 females; mean age = 19.62 years, *SD* = 1.30 years) was included in the final data analysis. Within the sample of undergraduate students, 4% of them reported their major in applied psychology; 7% of them in sports training; 10.3% of them in national traditional sports; 20.2% of them in social sports guidance and management and 58.5% of them in physical education.

### Measures

#### Mind-wandering

The 5-item Mind-Wandering Questionnaire (MWQ), which is on a 6-point Likert scale ranging from 1 (almost never) to 6 (almost always), was used to measure the levels of mind-wandering (Mrazek et al., 2013). All of these items were averaged as the MWQ score, with greater scores indicating higher levels of mind-wandering (e.g., “I mind-wander during lectures of presentations”). The Chinese version of MWQ has been validated (Luo et al., 2016). In Chinese sample, the fit indices for confirmatory factor analysis of the scale were as follows: χ^2^/*df* = 3.6, comparative fit index (CFI) = 0.98, the Tucker–Lewis index (TLI) = 0.93, root mean square error of approximation (RMSEA) = 0.06, standardized root mean square residual (SRMR) = 0.03, indicating MWQ had an adequate model fit. The Cronbach’s reliability (0.74) for the Chinese sample was satisfactory.

#### Mindfulness

Dispositional mindful awareness was assessed using Mindfulness Attention and Awareness Scale (MAAS), which contains 15 items and is rated on a 6-point Likert scale ranging from 1 (almost always) to 6 (almost never) (Brown and Ryan, 2003). All of these items were averaged as the MAAS score, with greater scores indicating higher levels of mindfulness (e.g., “I could be experiencing some emotion and not be conscious of it until sometime later”). The Chinese version of MAAS has been developed as a reliable and valid instrument to assess trait levels of mindfulness with adequate model fit for confirmatory factor analysis as follows: χ^2^/*df* = 2.69, CFI = 0.94, non-normed fit index (NNFI) = 0.93, and RMSEA = 0.079 (Deng et al., 2012). Cronbach’s alpha for the MAAS was 0.85, indicating good reliability.

#### Physical activity

The physical activity levels were gauged by the International Physical Activity Questionnaire Short Form (IPAQ), which assesses the time spent in different kinds of vigorous, and moderate physical activities, as well as walking and sedentary behaviors per week (Craig et al., 2003). The IPAQ scoring protocol is to generate the total Metabolic Equivalent Task (MET) scores by multiplying the total minutes spent on vigorous, moderate physical activities, and walking by 8, 4, and 3.3, and then adding them together (Craig et al., 2003). Finally, the total IPAQ scores were log-transformed so as to attain normal distribution. The Chinese version of IPAQ also had acceptable reliability with an intraclass correlation coefficient (ICC) of 0.79 (Macfarlane et al., 2007).

#### Flow

The dispositional flow was assessed by the Short Dispositional Flow Scale (S-DFS) (Copyright © 2002, 2009 by S. A. Jackson, we have made a license purchase from Mind Garden, Inc., and received permission to use S-DFS in current study), which consists of 9 items and uses a 5-point Likert scale ranging from 1 (never) to 5 (always) (Jackson et al., 2008, 2010). The averaged scores for each item of S-DFS were calculated, with higher scores indicating greater levels of dispositional flow (e.g., “I found the experience extremely rewarding”). The validity on Chinese version of S-DFS has been examined with adequate model fit for confirmatory factor analysis as follows: χ^2^/*df* = 2.49, CFI = 0.91, NNFI = 0.88, and RMSEA = 0.058 (Liu, 2010). Cronbach’s alpha for the S-DFS was 0.73, indicating satisfactory reliability.

#### Data analytic plan

Descriptive statistics and correlation analysis were conducted in SPSS. Multiple mediation analysis was performed in The PROCESS macro for SPSS, which was used to examine the multiple mediation model (model 4) (Hayes, 2017). Model 4 is a multiple mediator models, whose mediators are allowed to be operated in parallel. In this study, physical activity (M1) and mindfulness (M2), which were assumed to be the mediators in parallel, were proposed to mediate the relationship between mind-wandering (X) and flow (Y). Since the bias-corrected bootstrap has been demonstrated to generate the most accurate confidence intervals in prior studies (MacKinnon et al., 2004; Cheung and Lau, 2008), the present study would apply the boot-strapping method to determine the significance of mediation effects. And 5,000 bootstrap samples were employed to generate 95% bias-corrected CIs for the indirect effects, which indicate significant if zero does not fall within the CI.

## Results

### Descriptive statistics and correlation analysis

Table 1 presents the means and results of bivariate correlation analysis, which indicated that the associations between wandering mind, physical activity, mindfulness, and flow were significant in the expected directions, except that there was not any association between physical activity and mindfulness. Change in mind-wandering was significantly and inversely associated with the change in physical activity, mindfulness, and flow. Change in flow was also strongly and positively related to change in physical activity and mindfulness. In addition, there was no significant correlation between age and other variables.

**TABLE 1:** Descriptive statistics among key study variables (*n* = 429).

Variables	Mean	*SD*	1	2	3	4
1. Age (years)	19.62	1.30				
2. Wandering mind	3.19	0.78	0.08			
3. Physical activity^#^	3.77	0.29	–0.03	−0.14**		
4. Mindfulness	4.20	0.60	–0.02	−0.48***	0.07	
5. Flow	3.82	0.49	–0.04	−0.12*	0.16**	0.19***

^#^Physical activity logarithmic transformed. *p < 0.05; **p < 0.01; ***p < 0.001.

According to Csikszentmihalyi’s (1997) research, human beings can experience flow when performing almost any activity. In the current study, we classified and counted types of different activities based on what the participants filled in S-DFS. Except for four people who did not fill in the relevant activity content, the rest of the subjects reported dispositional flow experience when attending the following specific activities: 84.62% of subjects reported participating in physical exercises (namely, ball sports, track-and-field, swimming, gymnastics, fitness, and martial arts), 9.32% of them reported taking part in leisure and entertainment activities (namely, watching videos, playing games, listening to music, drawing, and chatting), 4.66% of them reported engaging in learning, and 0.47% of them reported participating in maintenance activities (namely, eating and driving).

### Multiple mediation analysis

The PROCESS macro for SPSS (model 4) was applied to examine the multiple mediation model. Model 4 is the multiple mediator model, whose mediators are assumed to arrange in parallel (Hayes, 2017). In the current study, we used multiple mediation analysis (model 4) to test whether physical activity and mindfulness had any significant mediation effects on the relationship between mind-wandering and flow. In this study, the supposed order of independent, mediator, and dependent variables were mind-wandering (independent variable) predicted physical activity or mindfulness (two multiple mediators), which predicted flow (dependent variable). Since the bias-corrected bootstrap has shown to generate the most accurate CIs in prior studies (MacKinnon et al., 2004; Cheung and Lau, 2008), such bootstrapping method was conducted to determine the significant mediation effects. And 5,000 bootstrap samples were applied to generate 95% bias-corrected CIs for the indirect effects, which indicate significant if zero do not fall within the CI.

**FIGURE 1:**
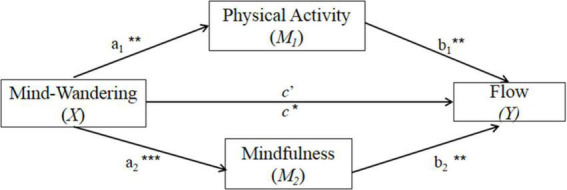
Mediational model of physical activity, mindfulness on mind-wandering and flow. *p < 0.05; **p < 0.01; ***p < 0.001.

Figure 1 and Table 2 present the multiple mediation model predicting flow in all the sample. Mind-wandering showed significant direct paths to the two mediators: physical activity (*a*_1_ = −0.053, *p* < 0.01) and mindfulness (*a*_2_ = −0.375, *p* < 0.001). Both mediated variables, physical activity (*b*_1_ = 0.243, *p* < 0.01) and mindfulness (*b*_2_ = 0.139, *p* < 0.01), revealed significant direct paths to flow. After controlling for the potential mediators of physical activity and mindfulness, mind-wandering showed no significant direct path to flow (*c*′ = −0.008, *p* = 0.817). The total effects of mind-wandering on flow were obtained by the sum of the direct and indirect effects: (*c* = *c*′ + *a*_1_ *b*_1_ + *a*_2_ *b*_2_ = −0.073, *p* < 0.05).

**TABLE 2:** Regression coefficients, standard errors, and model summary information for the parallel multiple mediator model depicted in Figure 1.

Antecedent	Consequent											
		*M*_1_ (PA)				*M*_2_ (M)				*Y* (F)		
		Coeff.	SE	T (*p*)		Coeff.	SE	T (*p*)		Coeff.	SE	T (*p*)
*X*(MW)	*a*_1_	−0.053	0.018	−2.963 (*p* < 0.01)	*a*_2_	−0.375	0.033	−11.402 (*p* < 0.001)	*c*′	−0.008	0.034	−0.231 (*p* = 0.817)
*M*_1_(PA)									*b*_1_	0.243	0.081	3.020 (*p* < 0.01)
*M*_2_(M)									*b*_2_	0.139	0.043	3.202 (*p* < 0.01)
Constant	*i*_M1_	3.940	0.058	67.496 (*p* < 0.001)	*i*_M2_	5.397	0.108	49.894 (*p* < 0.001)	*i*_Y_	2.341	0.406	5.764 (*p* < 0.001)
	*R*^2^ = 0.020 *F*(1, 427) = 8.778, *p* < 0.01				*R*^2^ = 0.233 *F*(1, 427) = 130.003, *p* < 0.001				*R*^2^ = 0.057 *F*(3, 425) = 8.499, *p* < 0.001			

MW, Mind-wandering; PA, Physical activity; M, Mindfulness; F, Flow; Coeff., Coefficient; SE, Standard error.

Table 3 shows the indirect effects and the related 95% CIs. In one pathway of “*Mind-wandering➔Physical activity➔Flow*,” this indirect effect (−0.0128) with a 95% bootstrap CI of −0.0283 to −0.0039 was significant. Another pathway of “*Mind-wandering➔Mindfulness➔Flow*,” whose indirect effect (−0.0522) with a 95% bootstrap confidence interval of −0.0881 to −0.0185 was significant. The total indirect effect [−0.0650 = (−0.0128) + (−0.0522)] was also significant with a 95% bootstrap CI of −0.1018 to −0.0302. Based on all these results, it showed the link between mind-wandering and flow was significantly mediated by physical activity and mindfulness.

**TABLE 3:** Indirect effects and 95% CIs.

Model pathways	Estimated	95% confidence interval	
		Lower	Upper
Total indirect effect	−0.0650*	–0.1018	–0.0302
Mind-wandering → physical activity → flow	−0.0128*	–0.0283	–0.0039
Mind-wandering → mindfulness → flow	−0.0522*	–0.0881	–0.0185
IndEff (physical activity) minus IndEff (mindfulness)	0.0394	0.0025	0.0770

*Empirical 95% CI does not overlap with zero. IndEff, Indirect effect.

In addition, effect contrasts (see Table 3) demonstrated that there was a significant difference in the two mediation effects between the indirect effects through physical activity and mindfulness [0.0394 = (−0.0128) to (−0.0522)], with a 95% bootstrap CI of 0.0025 to 0.0770. This indicated that mindfulness played a more important role than physical activity in the relationship between wandering-mind and flow.

## Discussion

The current study showed that physical activity and mindfulness significantly mediated the relationship between mind-wandering and flow, supporting the study hypothesis. Levels of mind-wandering correlated negatively with those of physical activity, mindfulness, and changes in flow correlated positively with changes in physical activity and mindfulness.

The negative correlation observed between mind-wandering and flow is consistent with previous findings, suggesting that these constructs can be viewed as opposites (Marty-Dugas and Smilek, 2018; Lambert and Csikszentmihalyi, 2019) and supporting the greater likelihood of individuals with fewer spontaneous thoughts to experience flow and address current tasks.

As mentioned previously, mind-wandering and flow have been reported to be closely correlated to creativity, respectively (MacDonald et al., 2006; Schooler et al., 2014; Williams et al., 2018; Schutte and Malouff, 2020), and our findings further confirmed that mind-wandering had a negative association with the flow. Further research is needed to examine the role of creativity in this relationship. Specifically, the identification of the types of spontaneous thought that facilitate and hinder the flow experience and creativity and the exploration of whether the flow is a mediator or a moderator of the relationship between mind-wandering and creativity, would be valuable.

As expected, mind-wandering predicted flow through the partial mediating effect of physical activity in this study. Nearly 85% of the participants in this study were engaged in physical exercise, related closely to the larger proportions of participants with sports- and physical education-focused majors. These results are consistent with previous reports of flow experiences mainly in the context of physical exercise (Jackson et al., 2008; Liu, 2010), and with the associated nature of increases in these two variables. Contrary to the present findings, mind-wandering was associated positively with moderate-to-vigorous physical activity in another study (Fanning et al., 2016); this difference may be due to differences in the measurement of mind-wandering and physical activity (a “Yes/No” question and accelerometers vs. the reliable and valid scales used in the current study). In future research, these tools should be applied concurrently to further clarify the relationship between mind-wandering and physical activity.

Notably, participants in this study reported experiencing flow not only during physical activity but also during engagement in leisure and entertainment activities, learning and maintenance activities (namely, eating and driving). Although individuals with flow experience feel in control and are deep enjoyment in their activities, such optimal experience is relatively rare in daily life (Csikszentmihalyi, 1997; Nakamura and Csikszentmihalyi, 2014). In most cases, an endless train of thought would stream through our mind, and be pervasive in our daily life (Smallwood and Schooler, 2015). We have reported the negative association between mind-wandering and flow, and physical activity and mindfulness might be the mechanism between mind-wandering and flow. The findings provide good insight into how daily spontaneous thought may affect the flow, which may improve the quality of life, and offer additional evidence to support the Context Regulation Hypothesis on mind-wandering (Smallwood and Andrews-Hanna, 2013; Smallwood and Schooler, 2015; Thomson et al., 2015; Smallwood et al., 2021).

Our findings further extend existing research by reporting the mediated effect of physical activity between mind-wandering and flow. People experiencing less mind-wandering are more likely to have higher levels of physical activity, enabling greater concentration on the information at hand, thereby bringing about more flow. More physically active people have been found to exhibit more satisfactory self-regulation (Buckley et al., 2014; Fanning et al., 2017), which enables them to more flexibly allocate executive and attentive resources and gain more sense of control over their thoughts, emotions, and behaviors. Our results are consistent with these findings. Moreover, physical activity as exercise breaks could induce less mind-wandering and enhance learning performance (Fenesi et al., 2018), suggesting that it facilitates the transformation of self-generated thoughts into goal-dependent processing and behavior. As mentioned previously, the spontaneous internal thoughts are dynamic, and mind-wandering is revealed as the adaptive attentional control to adjust the external environment (Schooler et al., 2011; Kam et al., 2013; Smallwood and Schooler, 2015). Our findings farther indicate that less mind-wandering might be related to have more attentional resources available to invest in the activity at hand and experience flow. And physical activity, as a beneficial means for promote attentional control and self-regulation, might be an important mechanism between mind-wandering and flow.

Our finding that mindfulness mediates the relationship between mind-wandering and flow is in line with previous studies that reported significant associations between mindfulness and wandering mind (Mrazek et al., 2012; Stawarczyk et al., 2012), and flow (Marty-Dugas and Smilek, 2018; Lambert and Csikszentmihalyi, 2019). Our participants’ MWQ scores reflect the detrimental aspects of mind-wandering, namely, interference with task execution. Mindfulness is an optimal strategy for the flexible regulation of attention (Mrazek et al., 2012; Stawarczyk et al., 2012) and the awareness of external and internal stimuli to modulate mind-wandering (Levinson et al., 2014; Schooler et al., 2014). As the task at hand progresses smoothly and attention resources are occupied by the relevant tasks, flow experience can be achieved through increased moment-to-moment attention (Nakamura and Csikszentmihalyi, 2014). On the other hand, self-generated thoughts about physical activity have been found to be related to more frequent participation in such activity, and thus constitute a positive form of mind-wandering that might stimulate physical activity (Rice and Fredrickson, 2017). And as mentioned earlier, successful self-regulatory physical activity could enhance executive functions, and contribute to flow experience (Jackson and Eklund, 2002; Elbe et al., 2010; Fanning et al., 2016). Thus, physical activity and mindfulness may both be influenced by spontaneous thoughts, and intervention involving them could be developed as strategies for thought regulation, and the promotion of concentration on the present moment, perhaps enabling the attainment of flow.

## Limitations and future implications

When interpreting the contributions of the current findings, the limitations and future implications of this study should be considered. First, the generalizability of the current findings is limited by the sample composition; the majority of the participating college students were majoring in fields involving motor learning and sports training, and thus might have higher physical activity levels and more flow experience than observed in general populations. In future studies, the current mediation models should be tested with samples of college students whose majors are less associated with physical activity. Second, the parallel multiple mediator model used in this study was established in an Eastern cultural context; its validity in other counties and cultural contexts should be examined. Third, the cross-sectional correlational design of the study prevented us from inferring causality. In future research, physical activity and mindfulness interventions should be developed based on the present findings, and their effects on the relationship between mind-wandering and flow should be examined longitudinally. Fourth, the roles of factors such as age, gender, and personality traits in the mediating effects observed in this study need to be investigated. Fifth, the MAAS provides a one-dimensional measure of mindfulness. Other scales, such as the Five Facet Mindfulness Questionnaire (FFMQ), could be used to assess different aspects of mindfulness (e.g., observing, describing, acting with awareness, non-judgment, and non-reaction) (Baer et al., 2006) and their impacts on the relationship with mind-wandering. Sixth, the scales used in the current study inevitably limited the reliability of our findings. In future research, the Experience Sampling Method should be used to dynamically assess mind-wandering, mindfulness, and flow in daily life, to confirm the findings obtained with the mediation models (Nakamura and Csikszentmihalyi, 2014; Smallwood and Schooler, 2015). Finally, although we found no significant relationship between dispositional mindfulness and physical activity, the two mediators identified in this study, a positive association between state mindfulness and physical activity was observed in a previous study in which the State Mindfulness Scale for Physical Activity was developed (Cox et al., 2016). Future studies should consider this issue with the application of this scale (Cox et al., 2016).

## Conclusion

The present study was aimed to investigate the relationships between mind-wandering, flow, physical activity, and mindfulness. We found a negative association between mind-wandering and flow, and the relationship was mediated by physical activity and mindfulness. The findings of this study have several practical implications. For instance, if individuals want to cultivate the flow in their daily life, they first need to decrease mind-wandering. Also, it raises the possibility that the development of physical activity and mindfulness can act as the preventive strategies to achieve flow.

## Author’s note

The authors received a license to use the Short Dispositional Flow Scale (S-DFS) (Copyright © 2002, 2009, S. A. Jackson) from Mind Garden, Inc. and received permission to use S-DFS in the current study.

## Data availability statement

The raw data supporting the conclusions of this article will be made available by the authors, without undue reservation.

## Ethics statement

The studies involving human participants were reviewed and approved by the Shanghai University of Sport, Shanghai, China; and Institute of Sports Science, Nantong University, Nantong, Jiangsu Province, China. The patients/participants provided their written informed consent to participate in this study.

## Author contributions

All authors listed have made a substantial, direct, and intellectual contribution to the work, and approved it for publication.
