# Corrigendum: The impacts of mind-wandering on flow: Examining the critical role of physical activity and mindfulness

**DOI:** 10.3389/fpsyg.2022.1019707

**Published:** 2022-09-21

**Authors:** Yu-Qin Deng, Binn Zhang, Xinyan Zheng, Ying Liu, Xiaochun Wang, Chenglin Zhou

**Affiliations:** ^1^Institute of Sports Science, Nantong University, Nantong, China; ^2^School of Psychology, Shanghai University of Sport, Shanghai, China; ^3^School of Kinesiology, Shanghai University of Sport, Shanghai, China

**Keywords:** mind-wandering, physical activity, mindfulness, flow, mediation analysis

In the published article a required Author's note was missing. The authors received a license to use Short Dispositional Flow Scale (S-DFS) from Mind Garden, Inc. which was not mentioned in the article. This has now been included in an Author's note as follows:

**Author's note**

The authors received a license to use the Short Dispositional Flow Scale (S-DFS) (Copyright © 2002, 2009, S. A. Jackson) from Mind Garden, Inc. and received permission to use S-DFS in the current study.

In addition, in the published article there were errors in Materials and methods, Measures, Flow, Paragraph 1. Firstly, Jackson, et al., 2010 was not cited. The citation has now been inserted in the revised paragraph below. Secondly, in sentence 1, the following statement was missing “(Copyright © 2002, 2009 by S. A. Jackson, the authors have made a license purchase from Mind Garden, Inc., and received permission to use S-DFS in the current study),” Finally, in sentence 2 the example given, “I feel I am competent enough to meet the high demands of the situation” should have been “I found the experience extremely rewarding”. The paragraph has now been revised as follows:

“The dispositional flow was assessed by the Short Dispositional Flow Scale (S-DFS) (Copyright © 2002, 2009 by S. A. Jackson, we have made a license purchase from Mind Garden, Inc., and received permission to use S-DFS in current study), which consists of 9 items and uses a 5-point Likert scale ranging from 1 (never) to 5 (always) (Jackson et al., 2008, 2010). The averaged scores for each item of S-DFS were calculated, with higher scores indicating greater levels of dispositional flow (e.g., “I found the experience extremely rewarding”). The validity on Chinese version of S-DFS has beenexamined with adequate model fit for confirmatory factor analysis as follows: *χ*^2^/df = 2.49, CFI = 0.91, NNFI = 0.88, and RMSEA = 0.058 (Liu, 2010). Cronbach's alpha for the S-DFS was 0.73, indicating satisfactory reliability.”

The authors apologize for these errors and state that this does not change the scientific conclusions of the article in any way. The original article has been updated.
